# MicroRNA-126 Inhibits Tumor Cell Growth and Its Expression Level Correlates with Poor Survival in Non-Small Cell Lung Cancer Patients

**DOI:** 10.1371/journal.pone.0042978

**Published:** 2012-08-10

**Authors:** Jiyun Yang, Haitao Lan, Xiaobing Huang, Baoyu Liu, Yu Tong

**Affiliations:** 1 Center for Human Molecular Biology and Genetics, Institute of Laboratory Medicine, Sichuan Academy of Medical Sciences and Sichuan Provincial People’s Hospital, Chengdu, Sichuan, China; 2 The Key Laboratory for Human Disease Gene Study of Sichuan Province, Sichuan Academy of Medical Sciences and Sichuan Provincial People’s Hospital, Chengdu, Sichuan, China; 3 Laboratory of Early Developmental and Injuries, West China Institute of Woman and Children’s Health, Sichuan University, Chengdu, Sichuan, China; 4 Key Laboratory of Obstetric and Gynecologic and Pediatric Diseases and Birth Defects of Ministry of Education, West China Second University Hospital, Sichuan University, Chengdu, Sichuan, China; 5 Department of Oncology, Sichuan Academy of Medical Sciences and Sichuan Provincial People’s Hospital, Chengdu, Sichuan, China; 6 Department of Hematology, Sichuan Academy of Medical Sciences and Sichuan Provincial People’s Hospital, Chengdu, Sichuan, China; 7 Department of Thoracic Surgery, Chengdu Army General Hospital, Chengdu, Sichuan, China; Queen Elizabeth Hospital, Hong Kong

## Abstract

**Background:**

It is controversial whether microRNA-126 is a tumor suppressive or oncogenic miRNA. More experiments are needed to determine whether microRNA-126 is associated with non-small cell lung cancer risk and prognosis.

**Methods:**

Over-expression of microRNA-126 was performed to evaluate the cell invasion and tumor growth in non-small cell lung cancer (NSCLC) cell lines and nude mouse xenograft model. Gain-of-function experiments and luciferase assays were performed to reveal the relationship between microRNA-126 and PI3K-Akt signal pathway in A549 cells. We analyzed the associations of the microRNA-126 expression between genetic variants within microRNA-126 and clinical information including smoking status, sex, age, and histological type and the tumor stage.

**Results:**

Over-expression of microRNA-126 in NSCLC cell lines decreased cell proliferation in vitro and tumor growth in the nude mouse xenograft model. And microRNA-126 repressed the activity of PI3K-Akt pathway by targeting binding sites in the 3′-untranslated region of PI3KR2 mRNA. The expression level of microRNA-126 was decreased in NSCLC lines and tumor tissues. The patients with low microRNA-126 expression had significantly poorer survival time than those with high microRNA-126 expression (means for survival time (month): 24.392±1.055 vs. 29.282±1.140, *P* = 0.005). However, there was no significant difference in the genotype and allele frequencies of the microRNA-126 variant (G>A, rs4636297) between cases and controls (*P* = 0.366). In addition, there was no association between SNP rs4636297 and survival time in NSCLC patients (*P* = 0.992). And microRNA-126 expression had no significant difference among the three genotype groups (*P* = 0.972).

**Conclusions:**

Our data indicate that microRNA-126 is a tumor-suppressor gene in NSCLC and low microRNA-126 expression is a unfavorable prognostic factor in NSCLC patients. However, the regulatory mechanism of microRNA-126 remains to be elucidated in different normal and malignant tissues. Therefore, further research is needed to explore the tumor suppressive functions of microRNA-126 in NSCLC.

## Introduction

Non-small cell lung cancer (NSCLC) remains the leading cause of cancer-related deaths in the world as well as in China [1]. A lot of genes involve in NSCLC tumorigenesis such as *p53*, *Rb*, and *Ras* [2]. A study shows that 98 of 186 microRNAs are in cancer-associated genomic regions or in fragile sites [3]. For example, miR-99a, let-7, miR-125b-2 are located in homozygous deletion regions without known tumor suppressor genes in lung cancer [3]. Several microRNAs are located near breakpoint regions, including miR-142 at 50 nucleotides from the t (8, 17) break involving chromosome 17 and MYC [3]. This indicates that microRNAs might play a crucial role in tumorigenesis and cancer progression [3]. In addition, microRNA expression profiling reveals characteristic signatures for many tumor types and is a biomarker of tumor classification, prognosis, and therapeutic outcome [4]–[8].

MicroRNA-126 is mapped to chromosome 9q34.3 within the host gene encoding epidermal growth factor like-7 (*EGFL-7*). MicroRNA-126 is highly expressed in lung and heart tissues but also expressed at lower levels in the brain, liver, and kidney [9]. High expression levels of microRNA-126 expression is identified on endothelial cells, which has an important role in regulating angiogenesis and blood vessel integrity by inhibiting the VEGF pathway. Knockdown of microRNA-126 resulted in loss of vascular integrity and hemorrhage during embryonic development of zebrafish[10]–[13]. Moreover, some studies revealed that microRNA-126 repressed apoptosis of acute myeloid leukemia cells and enhanced the colony-forming ability of mouse bone marrow progenitor cells through targeting Polo-like kinase 2 (*PLK2*) [14]. On the contrary, other studies showed that microRNA-126 is often referred to tumor suppressor gene in various cancers [15]–[21]. In gastric cancer, over-expression of microRNA-126 significantly enhanced the anchorage-dependent and anchorage-independent cell growth by targeting *SOX2* [16]. In colon cancer, microRNA-126 suppresses the growth of tumor cells by targeting phosphatidylinositol 3-kinase regulatory subunit beta (p85β) [19]. Some studies also show that microRNA-126 can inhibit invasion and proliferation by regulating *Crk*, *VEGF*, and *EGFL7* in NSCLC [15], [21], [22]. Thus, current evidences seem to support a close relationship between microRNA-126 and tumor growth. Moreover, compared to the patients not responding to the first line treatment with capecitabine and oxaliplatin, microRNA-126 expression level was significantly higher in patients responding to treatment with capecitabine and oxaliplatin [23]. In addition, the ratio of microRNA-126/microRNA-152 enabled the detection of urothelial bladder cancer (BCa) from urine at a specificity of 82% and a sensitivity of 72% [24]. Based on the above reports, microRNA-126 might have possible predictive and diagnostic value for cancers.

It is controversial whether microRNA-126 is a tumor suppressive or oncogenic miRNA. And the regulatory mechanism of microRNA-126 remains to be elucidated in different normal and malignant tissues [25]. Currently, more experiments are needed to determine whether microRNA-126 is associated with non-small cell lung cancer risk and prognosis. In this study, we explore the role of microRNA-126 in NSCLC and demonstrate that it is a tumor suppressor gene and its expression level correlates with poor survival in NSCLC patients.

## Results

### MicroRNA-126 Inhibits Cell Invasion Assay and Tumor Growth

**Figure 1:**
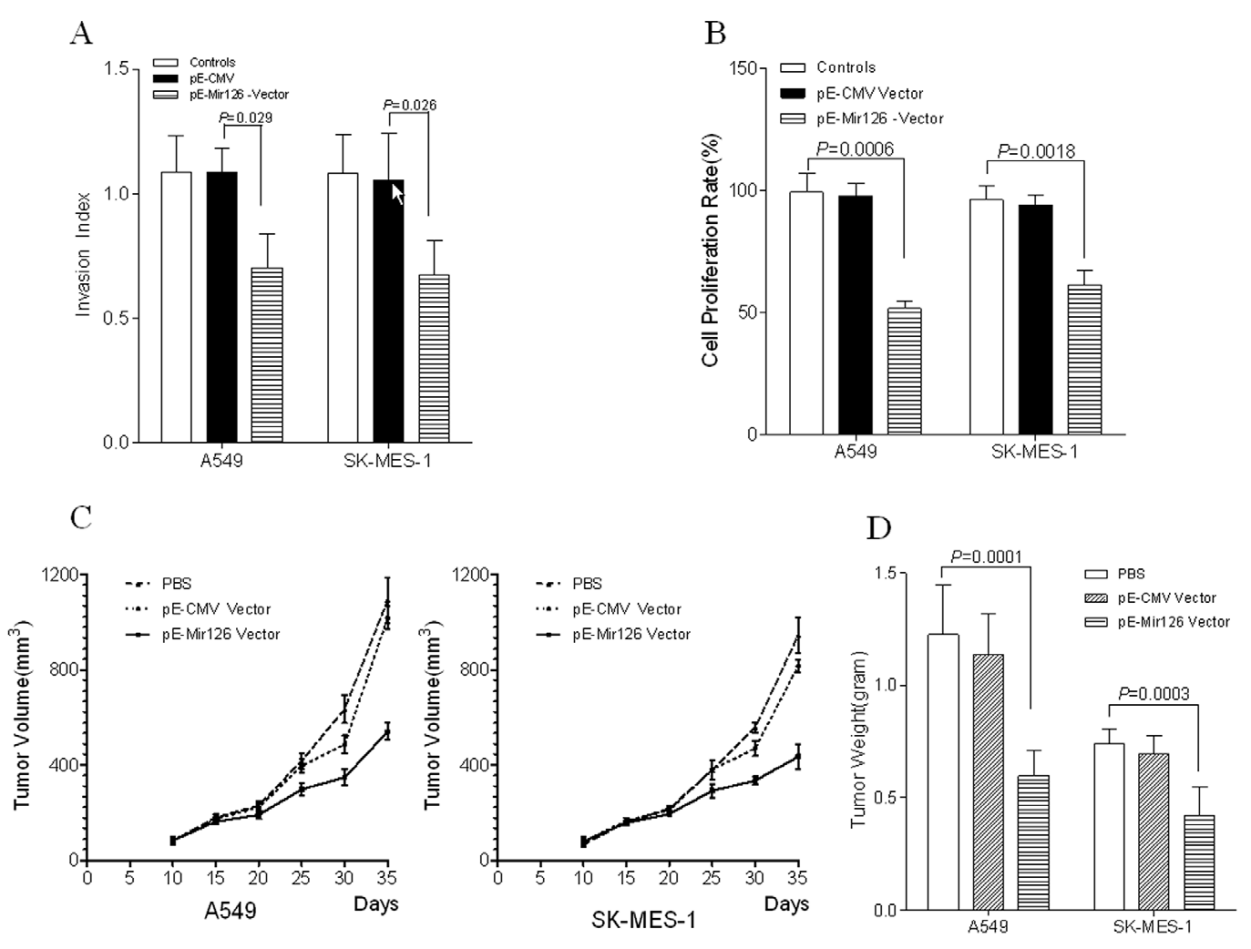
MicroRNA-126 inhibits cell invasion and tumor growth. (A) Overe-xpression of microRNA-126 inhibits the cell invasion in A549 and SK-MES-1 cells. Compared with the control group, over-expression of microRNA-126 impaires cell invasion. (B) MicroRNA-126 impairs the cell proliferation in A549 and SK-MES-1 cells. The cell proliferation was dramatically decreased after cells were treated with microRNA-126 over-expression for 72 hours. (C) The tumor growth curve *in vivo* by intratumoral injection with microRNA-126. The growth of tumors was observed from 1 to 25 days after the last injection. (D) MicroRNA-126 inhibits growth of A549 cell and SK-MES-1 cells in vivo. The average tumor only about an half of the tumors weight in the mice treated with PBS or pE-CMV vector alone.

We performed a cell invasion assay in the A549 and SK-MES-1 cells with pE-Mir126 vector or pE-CMV vector transfection. Compared with the control group, over-expression of microRNA-126 impaired cell invasion, which is consisted with previous reports (Figure 1A). Cell proliferation was decreased by 47.91% (*P* = 0.0006) and 36.36% (*P* = 0.0018), when respectively A549 and SK-MES-1 cells were respectively treated with microRNA-126 over-expression for 72 hours (Figure 1B). To further explore the effect of microRNA-126 on tumor growth, we investigated the ability of microRNA-126 to suppress tumor growth *in vivo* by intratumoral injection of pE-Mir126 vector into A549 and SK-MES-1 xenografts models of nude mice. As shown in Figure 1C, the growth of tumors was observed from 1 to 25 days after the last injection. The average tumor weight in the mice treated with pE-Mir126 vector at day 25 after the last injection was only about an half of the tumor weight in the mice treated with PBS alone or pE-CMV vector alone (Figure 1D). All tumors growth in the mice treated with pE-Mir126 vector were significantly suppressed compared to those in PBS-treated and pE-Mir126 vector treated mice.

### MicroRNA-126 Inhibits Tumor Cell Proliferation though PI3K-Akt Pathway in NSCLC

**Figure 2:**
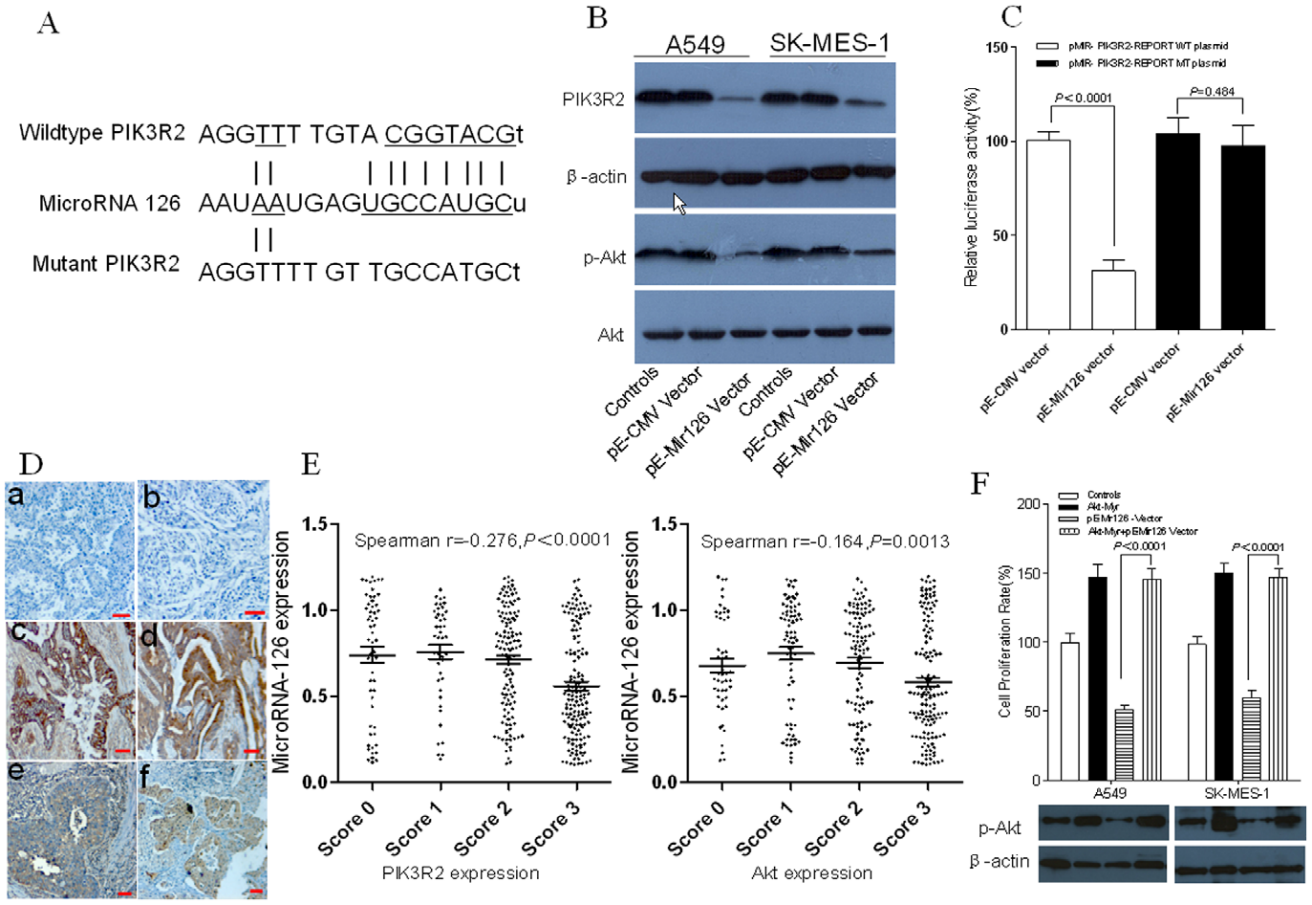
MicroRNA-126 inhibits the tumor cell proliferation though PI3K-AKT pathway in NSCLC. (A) Putative microRNA-126 targeting site in the 3′UTR of PIK3R2. Mutation was generated in the 3′UTR of PIK3R2 sequence in the complementary site for the seed region of microRNA-126 as indicated. (B) MicroRNA-126 binding site in the 3′UTR of PIK3R2 was assessed using the luciferase reporter assay. Luciferase activity was detected by a luminometer after co-transfection for 48 hours. The luciferase activity of each sample was normalized to the Renilla luciferase activity. (C) MicroRNA-126 suppresses PIK3R2 expression and Akt phosphorylation in A549 and SK-MES-1 cells. Total protein was isolated from cells treated with pE-Mir126 vector or pE-CMV vector for 48 hours. PIK3R2 expression and Akt phosphorylation were analyzed by western blotting using the antibody to P85β and phospho-Akt (Ser473). (D) The PIK3R2 expression levels and Akt phosphorylation in NSCLC tissues. Scale bar, 10 µm. a. Immunohistochemical staining negative control of PIK3R2 in NSCLC tissue. b. Immunohistochemical staining negative control of p-Akt (Ser473) in NSCLC tissue. c. Immunohistochemical staining of PIK3R2 in NSCLC tissue with low microRNA-126 expression levels. d. Immunohistochemical staining of p-Akt (Ser473) in NSCLC tissue with low microRNA-126 expression levels. e. Immunohistochemical staining of PIK3R2 in NSCLC tissue with high microRNA-126 expression levels. f. Immunohistochemical staining of p-Akt (Ser473) in NSCLC tissue with high microRNA-126 expression levels. (E) The expression levels of PIK3R2 and Akt phosphorylation in NSCLC tissues inversely correlated with the microRNA-126 expression levels (PIK3R2, Spearman r = −0.276, *P<*0.0001, Akt, Spearman r = −0.164, *P* = 0.0013). (F) Treatment with Akt-myr resulted in a statistically significant rescue of A549 and SK-MES-1 cell proliferation by transfection with pE-Mir126 vector. Total protein was isolated from cells treated with Akt-myr, pE-Mir126 vector or respective controls for 48 hours. Akt phosphorylation was analyzed by western blotting using the antibody to phospho-Akt (Ser473). After cells were transfected with Akt-myr, pE-Mir126 vector or respective controls for 72 hours in 96-well plate, cell proliferation rates were determined by the MTT assay.

To explore the molecular mechanism of microRNA-126 anti-proliferative effect on NSCLC, we used two open access programs (TargetScan and miRBase) to predict targets of microRNA-126. The predicted binding sites in the 3′UTR of *PIK3R2* for microRNA-126 are shown in Figure 2A. The luciferase reporter assay indicated that the activity of the reporter containing the 3′UTR of *PIK3R2* gene was decreased after co-transfection with pE-Mir126 vector (100.67±4.51 vs.31.33±5.86, *P<*0.0001), whereas the activity of site-directed mutagenesis of the reporter containing the 3′UTR of *PIK3R2* gene was not obviously altered (104.00±8.54 vs. 98.33±10.12, *P* = 0.484) (Figure 2B). In addition, western blot analysis revealed that the expression of PIK3R2 was impaired by treatment with pE-Mir126 vector in A549 (0.97±0.14 vs.0.31±0.07, P = 0.002) and SK-MES-1 cells (0.94±0.13 vs.0.45±0.08, *P* = 0.005) (Figure 2C). Furthermore, transfection of pE-Mir126 vector rather than pE-CMV vector significantly repressed Akt phosphorylation without changing the expression levels of total Akt protein (A549 cells, 0.89±0.10 vs.0.41±0.04, P = 0.002. SK-MES-1 cells 0.88±0.08 vs.0.47±0.05 *P* = 0.002) (Figure 2C).

To further evaluate the relationship between microRNA-126 and *PIK3R2* in NSCLC, we detected the expression levels of microRNA-126, PIK3R2 and Akt phosphorylation in 381 clinical samples by quantitative real time PCR (qRT-PCR) and **immunocytochemistry** (IHC) respectively. The expression levels of PIK3R2 and Akt phosphorylation in tumor tissues inversely correlated with the microRNA-126 expression levels (PIK3R2, Spearman r = −0.276, *P*<0.0001, Akt, Spearman r = −0.164, *P* = 0.0013, Figure 2D&E and Table 1). The results shown in Figure 2F demonstrated that treatment with recovering Akt activity resulted in the statistically significant inhibition of proliferation in A549 cells and SK-MES-1 cells by transfection with pE-Mir126 vector (*P*<0.0001).

**Table 1:** The expression levels of PIK3R2 and the phosphorylation levels of Akt in NSCLC tissues.

miR-126 levels^a^	expression of PIK3R2 protein				the phosphorylation of Akt			
	Score 0	Score 1	Score 2	Score 3	Score 0	Score 1	Score 2	Score 3
Low	21(11.2%)	13(6.9%)	51(27.1%)	103(54.8%)	29(15.4%)	24(12.8%)	44(23.4%)	91(48.4%)
High	37(19.2%)	31(16.1%)	73(37.8%)	52(26.9%)	21(10.9%)	50(25.9%)	68(35.2%)	54(28.0%)
Total	58(15.2%)	44(11.6%)	124(32.5%)	155(40.7%)	50(13.1%)	74(19.4%)	112(29.4%)	145(38.1%)

a Patients were divided into two groups with low and high microRNA-126 expression levels, based on their microRNA-126 expression levels: those with less than median of microRNA-126 expression levels and those with more than or equal to median of microRNA-126 expression levels (median: 0.654).

### Levels of microRNA-126 Expression Correlate with Poor Survival in Non-small Cell Lung Cancer Patients

**Figure 3:**
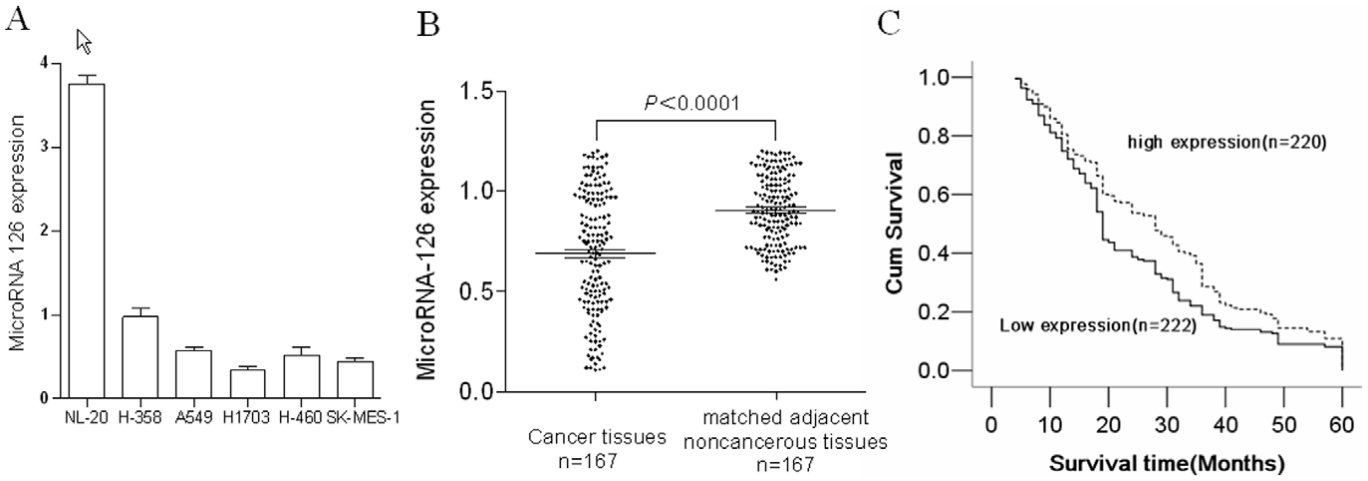
Low expression levels of microRNA-126 correlate with poor survival of NSCLC patients. (A) The expression levels of microRNA-126 are decreased in NSCLC cell lines. Expression of microRNA-126 was examined by quantitative real-time PCR in NL20 cell lines and NSCLC cell lines. (B) The expression levels of microRNA-126 are decreased in Human NSCLC specimens. Expression of microRNA-126 was determined by quantitative real-time PCR in tumor tissues and patient-matched adjacent lung tissues. Compared with the corresponding adjacent lung tissues, microRNA-126 expression was markedly down-regulated in tumor tissues (*P*<0.0001). (C) Low microRNA-126 expression correlates with poor survival of NSCLC patients. Patients were divided into two groups based on their microRNA-126 expression levels: those with less than median of microRNA-126 expression levels and those with more than or equal to median of microRNA-126 expression levels (median: 0.654). The patients with low microRNA-126 expression had significantly poor survival time compared with those with high microRNA-126 expression (means for survival time (month):24.392±1.055 vs. 29.282±1.140, *P* = 0.005).

Compared with the immortalized bronchial epithelial NL20, microRNA-126 expression levels were obviously decreased in NSCLC cell lines, such as A549, H358, H1703, H460 and SK-MES-1 (Figure 3A). The expression level of microRNA-126 was also examined in a cohort consisting of 168 pairs of NSCLC tumor tissues and matched adjacent noncancerous tissues (Figure 3B). Its expression level was much lower in the tumor tissues than that in noncancerous tissues (0.905±0.171vs.0.683±0.308, *P<*0.0001).

As depicted in Table 2, it was found that the expression levels of microRNA-126 did not correlate with sex, age, histological type, or the stage of tumor in the cohort of 442 NSCLC cases. Patients were divided into two groups based on their microRNA-126 expression levels: those with less than median of microRNA-126 expression levels and those with more than or equal to median of microRNA-126 expression levels (median: 0.654). However, Kaplan–Meier survival analysis revealed that the patients with low microRNA-126 expression level had significantly poor survival times compared with those with high microRNA-126 expression level (means for survival time (month):24.392±1.055 vs. 29.282±1.140, *P* = 0.005) (Figure 3C). Multivariate Cox proportional hazard regression analysis also showed that low microRNA-126 expression levels were a significantly unfavorable prognostic factor (hazard ratio, 0.782; 95% CI, 0.647 0.945) (Table 3).

**Table 2:** The expression levels of microRNA-126 in Non-small cell lung cancers.

Characteristic	No. of Patients	microRNA-126	*P* Value
Age (y)			0.961
<60	209	0.66±0.32	
≥60	233	0.65±0.31	
Gender			0.789
Male	341	0.66±0.31	
Female	101	0.67±0.35	
Smoking status			
Nonsmokers	125	0.69±0.32	0.387
smokers	317	0.66±0.32	
Histology			0.664
Squamous cell carcinoma	212	0.68±0.33	
Adenocarcinoma	208	0.65±0.32	
Other	22	0.65±0.27	
TNM staging			0.297
I	136	0.67±0.32	
II	146	0.68±0.30	
III	117	0.62±0.33	
IV	43	0.70±0.31	

**Table 3:** Clinical factors of patients correlate with overall survival by multivariate Cox proportional hazard regression analysis.

	*P* Value	hazard ratio	95% CI
Age	3.868×10^−6^	1.596	1.309–1.946
gender	0.182	1235	0.906–1.683
smoking	0.832	0.969	0.726–1.293
Histology	0.022	1.194	1.026–1.389
TNM staging	3.562×10^−24^	1.785	1.596–1.997
the level of has-mir-126	0.011	0.782	0.647–0.945
rs4636297	0.951	0.995	0.836–1.184

### Genetic Variant within MicroRNA-126 is not Associated with Cancer Risk and Survival Time in NSCLC Patients

**Figure 4:**
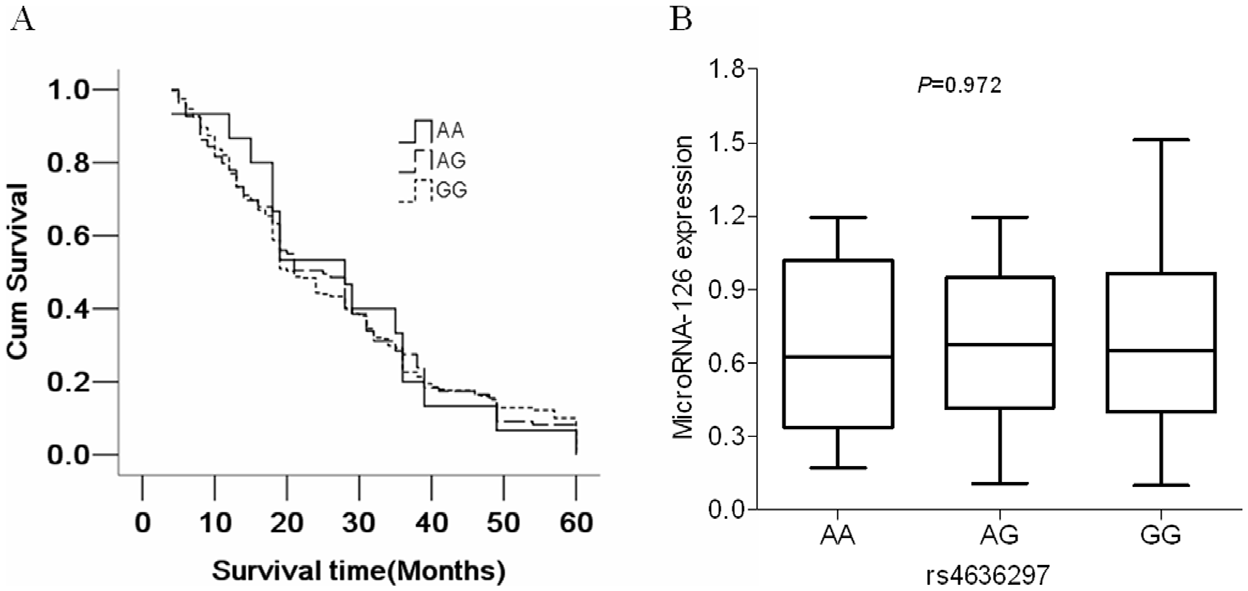
Genetic variant within microRNA-126 is not associated with the survival times and microRNA-126 expression levels in NSCLC patients. (A) Genetic variant within microRNA-126 is not associated with survival times. Kaplan-Meier survival estimates show that there is no association between SNP rs4636297 and survival time in NSCLC patients (*P* = 0.992). (B). Expression levels of microRNA-126 in NSCLC tissues of three genotypes are similar. MicroRNA-126 expression was determined by quantitative real-time PCR. There was no significant difference among the three genotype groups (*P* = 0.972).

We sequenced the genomic DNA segments including the pre-miR-126 and its respective flanking regions (±200 bp) in 442 NSCLC patients and 543 matched controls. Three SNPs, including rs78242242, rs4636297and rs1140713, were verified in the segments. In the present study, rs78242242 and rs1140713 were not statistically analyzed due to very low frequency (data not shown). The genotype distribution of the SNP (G>A, rs4636297) in the controls was accorded to the Hardy-Weinberg equilibrium (*P* = 0.336), and there was no overall difference in the genotype distributions between the cases and controls (Table 4). Kaplan-Meier survival estimates showed that there was no association between rs4636297 and survival time in NSCLC patients (*P* = 0.992) (Figure 4A). Multivariate Cox proportional hazard regression analysis also showed that rs4636297 was not associated with overall survival of NSCLC patients (hazard ratio, 0.995; 95% CI, 0.836–1.184) (Table 3). We also evaluated whether the rs4636297 polymorphism was associated with microRNA-126 expression in NSCLC and the results showed that there was no significant difference among the three genotype groups (*P* = 0.972) (Figure 4B).

**Table 4:** Genotype of microRNA-126 polymorphisms and their associations with NSCLC risk.

Genotypes	Cases (N = 442)	Controls (n = 543)	*P* value	Adjusted OR (95% CI)	*P* trend
GG	0.719(318)	0.678(368)	0.366	1.000	0.183
AG	0.247(109)	0.284(154)		1.136(0.557–2.315)	
AA	0.034(15)	0.039(21)		1.282(0.945–1.739)	
A allele	0.157(139)	0.180(196)	0.172		

## Discussion

In the past few years, it has been identified that the aberrant expressions of microRNAs play an important role in tumor formation and development [26]–[28]. In tumorigenesis, the expression levels of some microRNAs are decreased in cancer tissues. These types of microRNAs are considered as tumor suppressor genes. Tumor suppressor microRNAs usually prevent from oncogenesis by negatively regulating oncogenes or genes that control cell proliferation, differentiation, migration and apoptosis [29], [30]. Currently, microRNA-126 expression level markedly decreases in various tumor tissues, including NSCLC, colon cancers, breast cancer and gastric cancer [16]–[22], [31]. Therefore, microRNA-126 is considered as tumor suppressor genes.

Our data showed that over-expression of microRNA-126 impaired NSCLC cell proliferation and tumor growth in the xenografts model of nude mice. It is generally accepted that miRNAs exert their function by downregulating the expression of their downstream target genes. Activation of the PI3K pathway results in cell growth and survival, which contributes to tumor growth, metastatic and therapy resistance in NSCLC [32]. Several studies have indicated that the PI3K-Akt pathway plays a central oncogenic role in inducing cell proliferation and tumor development [33], [34]. This pathway is therefore an attractive target for the anticancer agents. Based on bioinformatics analysis for prediction targets of microRNA-126, the *PIK3R2* gene was selected for the potential targets of microRNA-126. In this study, we found that *PIK3R2* gene was a direct target of microRNA-126. Furthermore, microRNA-126 expression in both NSCLC cell lines and tumor tissues markedly decreased compared with noncancerous cells and tissues. Our data aslo showed that over-expression of microRNA-126 impaired NSCLC cell proliferation and tumor growth in A549 xenografts model of nude mice though regulation of PI3K-Akt signaling pathway. These data suggested that the loss of microRNA-126 expression may involve the development and progression of NSCLC. Consistent with these past findings [22], our results also showed that expression levels of microRNA-126 expression correlate with poor survival in non-small cell lung cancer patients.

Genetic variants in precursor microRNA (pre-miRNA) or miRNA target sites have been shown to be associated with the risk of various cancers. Some studies show that SNPs in pre-miRNAs played an important role in the prediction of NSCLC survival and susceptibility [35], [36]. For example, miR-196a2 variant homozygotes had a 1.76-fold elevated hazard ratio (HR) for an unfavorable overall survival of NSCLC [36]. However, no polymorphisms were found in the predicted binding sites in the 3′UTR of PIK3R2 for microRNA-126. So we performed the genetic association analysis to study potential association of SNPs within microRNA-126 with survival and susceptibility of NSCLC. In the present study, we found that the genotype and allele frequencies of the microRNA-126 (G>A, rs4636297) SNPs were not associated with the risk and overall survival of NSCLC. In previous report, the association between the rs4636297 and breast cancer risk has not been found [37]. Recent study demonstrates that rs4636297GG genotype significantly blocks the processing of pri-miRNA to pre-miRNA, resulting in the significantly reduced mature microRNA-126 expression [38]. Similarly, compared to the wild-type G allele, the A variant of rs4636297 has no effect on the secondary structure of pre-miR-126 itself, but it affects the secondary structure of flanking region. Meanwhile, the free energy ΔG is increased in the A allele variant [37]. However, microRNA-126 level had no significant difference among the three genotype groups in our cohort of 442 NSCLC cases. The results indicate that gremlins variants in the microRNA-126 have no impact on the microRNA level of tumor tissues in NSCLC patients. Although a study identified that Ets-1 and Ets-2 can regulate the expression of microRNA-126 by targeting an Ets binding element in genomic regions upstream of the microRNA-126 and *EGFL7* gene in endothelial cells [39], further studies are necessary to determine whether the above mechanism involves in the down-regulation of microRNA-126 in NSCLC.

In summary, our data indicate that microRNA-126 is a tumor-suppressor gene in NSCLC and low microRNA-126 expression is a significantly unfavorable prognostic factor in NSCLC patients. However, the genetic variants of microRNA-126 are not associated with NSCLC risk and prognosis. In addition, the regulatory mechanism of microRNA-126 remains to be elucidated in different normal and malignant tissues. Therefore, further research is needed to explore the tumor suppressive functions of microRNA-126 in NSCLC.

## Materials and Methods

### Cell Culture, Study Population and Surgical Specimens

A549, H358, H1703, H460 and SK-MES-1 human NSCLC lines, a normal human bronchial epithelial cell line (NL-20), were obtained from the American Type Culture Collection. Human NSCLC specimens were from 442 patients at Chengdu Army General Hospital and Sichuan Provincial People’s Hospital from 2008 and to 2010. Of 442 specimens, 76 were obtained by biopsy from 43 cases of stage IV and 33 cases of stage III, and the rest obtained by surgery. There are 168 adjacent noncancerous lung tissues in the 442 specimens. The stage of a NSCLC is based on the American Joint Committee on Cancer (AJCC) TNM System. Cancer-free controls were from a cohort of 543 individuals who had no history of cancer and were matched to the cases on age and sex. This study was approved by the Institutional Review Board of Sichuan Academy of Medical Sciences & Sichuan Provincial People’s Hospital, West China Second University Hospital, Sichuan University and Chengdu Army General Hospital. The signed informed consent was obtained from all participants or from patients’ representatives if direct consent could not be obtained. None of the patients had received any treatment for lung cancer before surgery. Study participants underwent a personal interview to obtain information on demographic traits and lifestyle factors, such as tobacco use.

### RNA Extraction and Quantitative Real Time PCR

Total RNA was extracted using TRIzol reagent. Total RNA (100 ng) of each sample was used for cDNA synthesis using reverse transcription primers for microRNA-126 and U6 small nuclear RNA. Quantitative real time PCR was performed using the SYBR® Premix Ex Taq™ (TaKaRa Biotech Co.). qPCR Primer Set for microRNA-126 (Cat.no.MQP-0101) and U6 small nuclear RNA (Cat.no.MQP-0201) were from Ribobio Co. MicroRNA-126 and U6 small nuclear RNA were respectively quantified according to standard curve and performed in triplicate. U6 small nuclear RNA was used for normalization. The relative expression was calculated using the equation: copies (miR-126)/copies (U6).

### Cell Invasion Assay

A transwell cell culture chamber (Millipore, Bedford, MA, USA) was coated with Matrigel, dried and reconstituted with culture medium at 37°C. Cells were divided into three groups: the control group, the pE-CMV group and the pE-Mir126 group. Cells transfected with pE-CMV or pE-Mir126 were starved in serum-free medium and suspended at 1×10^6^/ml in RMPI1640 medium for 24 hours prior to assay. 300 µl of cell suspension was placed in the upper chamber and allowed to migrate for 24 hours at 37°C. The suspended media in the lower chamber were removed. The cells that had invaded the lower side of the filter were fixed in 4% paraformaldehyde and stained with Gimsa solution. The number of cells that passed through the pores into the lower chamber was counted under a phase-contrast microscope.

### Cell Proliferation Assays

Cells were seeded onto 96-well plate in RMPI1640 with 10% FBS and 100 µg/ml penicillin/streptomycin for 72 hours. Cell proliferation rates were determined by the 3-(4, 5-dimethylthiazolyl-2)-2, 5-diphenyltetrazolium bromide (MTT) assay.

### Vector Constructs

The fragment 398 bp in length containing 3′UTR of *PIK3R2* was amplified by PCR and cloned into pMD-18T vector (Takara) using the following primers: forward, 5′- ACTAGTTGCAGATTCAGGGCTTCTCT -3′, and reverse, 5′-AAGCTTCCTGTATGACCTTGGGCACT -3′. Then, the fragment was subcloned into the Spe I and Hind III sites downstream of luciferase gene in the pMIR-REPORT plasmid (Ambion) to generate pMIR- PIK3R2-REPORT WT plasmid. Using pMIR- PIK3R2-REPORT WT plasmid as a template, pMIR- PIK3R2-REPORT MT plasmid, which carried the mutated *PIK3R2* 3′-UTR sequence in the complementary site for the seed region of microRNA-126, was generated by a KOD -Plus-Mutagenesis Kit (Toyobo, Japan) according to the manufacturer’s protocol. The following primers were used: forward, 5′-TGCCATGCTTGTTATTGATATGATATAAAACATC -3′, reverse, 5′-ACAAAACCTGCCTCCCAGCTCGTGGGGC -3′. All constructs were confirmed by sequencing.

### Luciferase Reporter Gene Assay

A549 cells were cultured in 96-well plates and cotransfected with pMIR- PIK3R2-REPORT WT plasmid (or pMIR- PIK3R2-REPORT MT plasmid) and pE-Mir126 vector (GeneSil Biotechnology Co.,Ltd,China) using lipofectamine 2000. Firefly and Renilla luciferase activities were measured 48 hours after transfection using the Dual Luciferase Reporter Assay kit (Promega, USA) according to the manufacturer’s protocol. The assays were performed in duplicate and repeated three times.

### Western Blot

The cells were lysed using RIPA buffer. Total protein (20 µg) was run on 12% SDS-PAGE and subsequently transferred onto polyvinylidene difluoride (PVDF) membrane. The membrane was blocked in tris-buffered saline tween-20 (TBS-T) with 3% BSA for an hour at room temperature. Then they were incubated with the primary antibodies overnight at 4°C and followed by incubation with the HRP-conjugated secondary antibodies for an hour at room temperature. Immunoreactive bands were identified using SuperSingal west pico chemiluminescent substrate and exposed to X-rays films.

### Immunocytochemistry

Immunohistochemical staining was performed on 5-mm sections of paraffin-embedded NSCLC tissues to determine the expression of PIK3R2 and Akt phosphorylation. Briefly, for blocking of endogenous peroxidase, the slides were incubated for 10 min with 3% H_2_O_2_, washed with PBS for 5 minutes, and then for 30 minutes with 3% goat serum in PBS. The slides were incubated in PIK3R2 and phospho-Akt (Ser473) antibody (1∶250, Cell signal technology) overnight at 4°C. Subsequent steps were performed using EnVision™ Systems (DAKO). The staining intensities are scored and represented as follows: Score 0: completely negative samples; Score 1: samples with up to10% of positive cells; Score 2: samples with 11–50% of positive cells; Score 3: samples with 50% of positive cells.

### Nude Mouse Xenograft Model

Female BALB/c nu/nu mice (4–5 weeks old) were purchased from Institute of Experimental Animals, Sichuan Provincial Academy of Medical Sciences (Chengdu, China). A549 cells (1×10^6^) were suspended in 100 µl PBS and injected subcutaneously in the right flank region of nude mice. After 10 days, the tumors reached 5–10 mm in diameter, and mice were randomly divided into three groups (5 mice per group), Animals received intratumoral injections of pE-Mir126 vector, pE-CMV vector or PBS, respectively for four times at days of 1, 3, 5 and 7, with the total dose of 3×10^10^ plaqueforming units (p.f.u.) per tumor. Tumor dimensions were measured 2 times every 5 days by a linear caliper. The tumor volume (mm^3^) was calculated according to the following formula: length × width^2^/2. All mice were sacrificed humanely on the thirtieth day after treatment, and the resected tumors were weighed.

### Genotyping

Variant rs2297882 was genotyped by sequencing. MicroRNA-126 including pre-miRNAs were amplified by PCR, The following primers were used to amplify the product: forward, 5′- ATTGCCGTGTGGCTGTTAG-3′; reverse, 5′- CATTGCACTGTCCACTCCTG -3′. PCR products were sequenced in both directions on an ABI3130xl Genetic Analyzer (Applied Biosystems, USA). The primers for PCR were also used as sequencing primers.

### Statistics Analysis

The level of microRNA-126 in different groups was evaluated by T test. The associations between NSCLC risk and SNP genotypes were estimated by multivariable logistic regression analyses. Survival was calculated from the date of initial diagnosis to the date of either death or last follow-up. Survivals were analyzed using the Kaplan-Meier method, and differences in distribution were evaluated by means of the log-rank test. The Cox proportional hazards model was applied to the multivariate analysis. *P* value <0.05 was defined as statistically significant. All results were analyzed by statistical software SPSS13.0.
